# A Fiber-Integrated CRDS Sensor for In-Situ Measurement of Dissolved Carbon Dioxide in Seawater

**DOI:** 10.3390/s21196436

**Published:** 2021-09-27

**Authors:** Mai Hu, Bing Chen, Lu Yao, Chenguang Yang, Xiang Chen, Ruifeng Kan

**Affiliations:** 1 Anhui Institute of Optics and Fine Mechanics, Hefei Institute of Physical Science, Chinese Academy of Sciences, Hefei 230031, China; humai@aiofm.ac.cn (M.H.); bchen@aiofm.ac.cn (B.C.); lyao@aiofm.ac.cn (L.Y.); xchen@aiofm.ac.cn (X.C.); 2 University of Science and Technology of China, Hefei 230026, China; 3 Institute of Deep-Sea Science and Engineering, Chinese Academy of Sciences, Sanya 572022, China; yangcg@idsse.ac.cn

**Keywords:** seawater dissolved gas, carbon dioxide, optical cavity ring-down spectroscopy, in-situ measurement

## Abstract

Research on carbon dioxide (CO_2_) geological and biogeochemical cycles in the ocean is important to support the geoscience study. Continuous in-situ measurement of dissolved CO_2_ is critically needed. However, the time and spatial resolution are being restricted due to the challenges of very high submarine pressure and quite low efficiency in water-gas separation, which, therefore, are emerging the main barriers to deep sea investigation. We develop a fiber-integrated sensor based on cavity ring-down spectroscopy for in-situ CO_2_ measurement. Furthermore, a fast concentration retrieval model using exponential fit is proposed at non-equilibrium condition. The in-situ dissolved CO_2_ measurement achieves 10 times faster than conventional methods, where an equilibrium condition is needed. As a proof of principle, near-coast in-situ CO_2_ measurement was implemented in Sanya City, Haina, China, obtaining an effective dissolved CO_2_ concentration of ~950 ppm. The experimental results prove the feasibly for fast dissolved gas measurement, which would benefit the ocean investigation with more detailed scientific data.

## 1. Introduction

Marine carbon cycling is the result of a series of physical, geological and biological processes on a spatiotemporal scale [1,2]. The ocean absorbs one-third of the anthropogenic carbon emission, about 2 billion tons per year. As such, the ocean becomes one important place for carbon sequestration [3]. Furthermore, CO_2_ is the main greenhouse gas, the main dissolved gas of seawater and the main fluid component of the deep-sea extreme window of cold spring and hydrothermal solution. Precise measurement on its spatiotemporal distribution is significant to investigate the biogeochemical material cycle and global climate change [4,5]. However, common methods based on sampling-laboratory analysis are not enough to support modern marine science. In-situ CO_2_ sensors with high sensitivity, high fidelity, large dynamic range and fast response are highly needed in many cutting-edge research topics, such as the sea-air exchange flux of CO_2_ [6], CO_2_ concentration of deep-sea cold spring and hydrothermal fluid components [7,8,9], and isotope measurement [7,8,10,11].

Currently, optical technology, semiconductor gas sensing and mass spectrometry [8,9,11,12,13] are common methods in in-situ measurement of dissolved gas in seawater. Among them, laser-based in-situ optical spectrometer is suitable for greenhouse gas sensing in seawater due to its unique selectivity and sensitivity [12,14,15]. Using infrared spectroscopy technology, the German Hydro C company demonstrated dissolved CH_4_ measurement in seawater [16]. Using the off-axis integrated cavity output spectroscopy (OA-ICOS), the LGR Company in the United States measured the dissolved CH_4_/CO_2_ and its isotope δ^13^CH_4_ in seawater [11]. Using mid-infrared absorption spectroscopy technology, Zheng Chuantao et al. achieved the measurement of dissolved CO_2_ and its isotope δ^13^CO_2_ [12]. In addition, cavity ring-down spectroscopy (CRDS), proposed by O, Keefe and Deacon in 1988 [17], has ultra-high detection sensitivity, light intensity jitter immunity and free instrument calibration, making CRDS one of the best candidates for gas detection. The past years have witnessed its remarkable progresses in precision spectroscopy [18,19,20] and important applications in atmospheric trace gas measurement [21,22,23]. However, the application of this technology in the marine field for in-situ measurement remains unresolved due to the challenges of high stability resonant cavity, high precision/low power circuit and time-consuming dissolved gas concentration retrieval.

In this paper, we report the development of an in-situ CRDS based dissolved CO_2_ sensor. A fast exponential regression model is proposed to retrieve the concentration of dissolved gas in seawater. In the implementation, we design high-pressure assembling and use polydimethylsiloxane (PDMS) membrane for water/gas separation and enrichment [24,25]. A long-time in-situ observation near the coast is carried out to prove the feasibility of in-situ separation, enrichment and measurement of dissolved CO_2_ in seawater.

## 2. Principle of CRDS-Based Seawater Dissolved Gas Measurement

### 2.1. Water/Gas Separation and Enrichment, and Dissolved Gas Retrieval

A PDMS membrane is one common tool, as the gas-liquid interface with a thickness of l, to separate and enrich seawater dissolved gases [26,27]. The concentration difference between both sides enables the dissolved gas pass through the membrane and blocks liquid water molecule (H_2_O)_n_. Thus, small gas molecules, such as CH_4_, CO_2_ and O_2_ can be separated from seawater. The water/gas separation of PDMS membrane is typically described by the “dissolution-diffusion” model [24,26]. When concentration difference between both sides exists, gas molecules diffuse into the membrane and realize gas exchange.

In the case of a stable situation, the dissolved gas concentration remains stable along the direction of film thickness. The diffusion flux on the side of the gas chamber can be expressed by Fick’s first law [24,26,27]:

(1) $$F_{G}=\frac{D_{G}S_{G}A(P_{G1}-P_{G2})}{l},$$

where $F_{G}$ (cm^3^·cm^2^(cm^2^polymer)^−1^·s^−1^) is the diffusion flux of gas component *G* per unit time, $D_{G}$ (cm^2^·s^−1^) is the diffusion coefficient of gas component *G* in the membrane, $S_{G}$ (cm^3^·(cm^2^polymer)^−1^·Pa^−1^) is the solubility coefficient of gas component *G* in the membrane, $P_{G1}$ (Pa) is the partial pressure of seawater dissolved gas *G*, $P_{G2}$ (Pa) is the partial pressure of gas component *G* in the gas chamber, *A* (cm^2^) is the film area, *l* (cm) is the film thickness. While gas diffusion flux can be expressed by Fick’s second law in the case of unstable situation [25,26]:

(2) $$F_{G,t}=F_{G,ss}\left(1+2\sum_{n=1}^{\infty }{\left(-1\right)}^{n}\exp\left\{\frac{-n^{2}\pi^{2}D_{G}t}{l^{2}}\right\}\right),$$

where, $F_{G,t}$ is the gas flux at time *t*, $F_{G,ss}$ is the gas flux in the stable situation.

The diffusion coefficient of CO_2_ in PDMS membrane is about 1.5 × 10^−5^ cm^2^/s, *l* ≪ 1 cm, *t* = 1 s, then $\exp\left\{\frac{-n^{2}\pi^{2}D_{G}t}{l^{2}}\right\}$ is almost zero and $F_{G,t}$ ≈ $F_{G,ss}$. Thus, the diffusion flux in this case can also be described by Fick’s first law. Therefore, the concentration change of the gas component *G* is as follows:

(3) $$P_{G2}*V=\int^{\;}\frac{D_{G}S_{G}A(P_{G1}-P_{G2})}{l}d_{t},$$

where *V* is the volume of the gas chamber. From Equation (3), we can obtain:

(4) $$\frac{d_{P_{G2}}}{d_{t}}*V=\frac{D_{G}S_{G}A(P_{G1}-P_{G2})}{l},$$

With the boundary condition, $t\to \infty ,P_{G2}=P_{G1}$, it will be extrapolated that:

(5) $$P_{G2}=K*\exp\left(-\frac{D_{G}S_{G}A}{lV}t\right)+P_{G1},$$

where *K* is related to the initial pressure. For non-condensable gases, such as CO_2_ and CH_4_, the value of $-\frac{D_{G}S_{G}A}{lV}$ is independent from partial pressure.

After measuring the value of $P_{G2}$ over time in an unbalanced situation, the exponential regression is used to retrieve $P_{G1}$. $P_{G1}$ equals to $P_{G2}$ in balanced situation.

### 2.2. Optical Measurement Using CRDS

When laser with an intensity of *I_in_* passes through uniform gas substance, the laser decays to *I_out_* due to gas absorption, which can be described by the Beer-Lamber law [10,17]:

(6) $$I_{out}=I_{in}*\exp\left(-\alpha *L\right),$$

where, *α* (cm^−1^) is the spectral absorption coefficient, *L* (cm) is the interaction distance.

**Figure 1:**
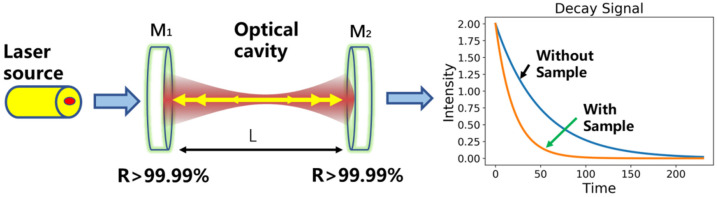
Schematic of the cavity ringdown technique. M_1_ and M_2_: cavity mirrors with high reflectivity (>99.99%).

In the configuration of CRDS, a couple of high reflectivity mirrors, usually higher than 99.99%, enable a significant interaction length extension inside a limited physical space [28], and become capable of detecting minor absorption of trace gas concentration. Its working principle is shown in Figure 1. When laser beam resonates with one resonant cavity mode, the laser power inside the cavity will be rapidly built up. With a trigger that the inside laser power reaches a certain threshold, the incident laser is quickly cut off to generate a free intracavity ring down. The leaked laser intensity $I_{\nu }\left(t\right)$ after each ring down is successively recorded at the exit to obtain optical intensity that decays with time as follows,

(7) $$I_{\nu }\left(t\right)=I_{0\nu }*\exp\left(-\frac{tc}{L_{mirrors}}\left(1-R+\alpha_{\nu }L_{mirrors}\right)\right),$$

where, *c* is the speed of light, *t* (μs) is the time, *L_mirrors_* (cm) is the physical distance between the two mirrors, *R* is the reflectivity of the optical cavity mirror, $\alpha_{\nu }$ (cm^−1^) is the spectral absorption coefficient of the specific wavelength; $I_{0\nu }$ is the initial laser intensity when the laser is cut off.

The time for the initial laser intensity reduces to 1/e in the measurement is defined as the ring-down time [17]. According to Equation (7), in the case of no gas absorption, the empty cavity ring-down time $\tau_{0}$ is:

(8) $$\tau_{0}=\frac{L_{mirrors}}{C\left(1-R\right)},$$

In the presence of gas absorption, the ring-down time $\tau_{\nu }$ is:

(9) $$\tau_{\nu }=\frac{L_{mirrors}}{C\left(1-R+\alpha_{\nu }L_{mirrors}\right)},$$

Combining (8) and (9), we obtain the intracavity spectral absorption coefficient as:

(10) $$\alpha_{\nu }=\frac{1}{C\tau_{\nu }}-\frac{1}{C\tau_{0}},$$

In the absorption spectrum [29], the absorption coefficient can be expressed as:

(11) $$\alpha_{\nu }=S\left(T\right)*P_{total}*X*\psi \left(\nu \right),$$

where *S*(*T*) (cm^−2^·atm^−1^) is the intensity of the absorption line, $P_{total}$ (atm) is the total pressure of the gas, *X* is the molecular concentration, *ψ*(*ν*) (cm) is the absorption line shape function.

Since integral of *ψ*(*ν*) over *ν* is equals 1, i.e., $\int_{-\infty }^{+\infty }\psi \left(\nu \right)d_{\nu }=1$, *S*(*T*) relates to the temperature for a specific absorption line, and $P_{total}$ can be measured using a commercial pressure sensor. Therefore, after fitting the absorption coefficient curve to obtain the integrated area *A*, the partial pressure of the gas can be calculated by:

(12) $$P=XP_{total}=\frac{A}{S\left(T\right)},$$

Thus, the partial pressure of dissolved gas ($P_{G1}$) can be retrieved by measuring the partial pressure of gas ($P_{G2}$) in the cavity and fitting formula (5).

## 3. In-Situ Dissolved CO_2_ Sensor Configuration

**Figure 2:**
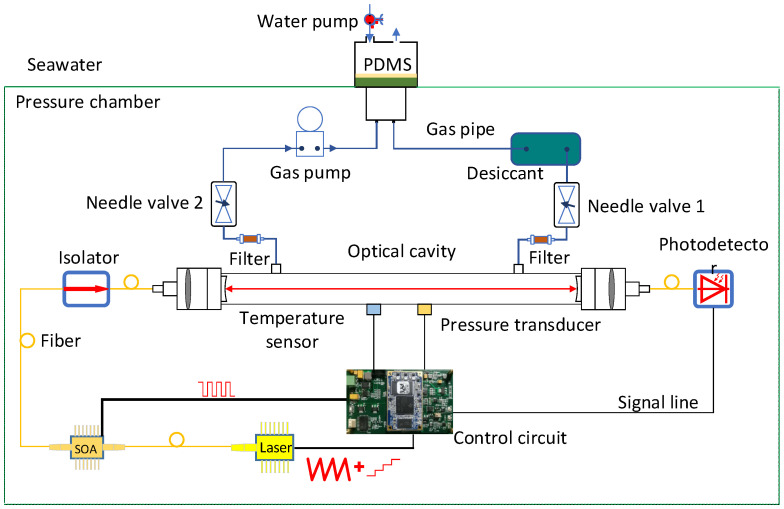
Framework of in-situ underwater instrument for dissolved gas measurement.

Figure 2 depicts the schematic diagram of the optical dissolved-CO_2_ sensor, which comprises three parts, one pressured chamber, one water/gas separation and enrichment unit and one gas measurement unit. The first part, i.e., the pressure chamber, is a dry titanium alloy chamber with an inner diameter of 128 mm, a length of 750 mm and a wall thickness of 10 mm. It can withstand a pressure as high as 57 MPa, which is suitable for experiments at about 4500 m under water. The second part, i.e., the water gas separation and enrichment unit consists of a water pump (SEA-BIRD SBE-5T), a PDMS membrane module (membrane thickness 50 μm, diameter 5 cm, gas separation efficiency 0.034 mL/min at 296 K and 1 atm pressure difference), a drying box, an air pump (KNF NMP05), a filter (Swagelok, SS-2F-05), two needle valves (Swagelok, SS-ORS2) and the gas chamber (optical resonant cavity). With the water pump, the seawater continuously flows through the surface of PDMS membrane at a flow rate of 0.8 L/min, forming a stable dissolved gas concentration field on the surface of the membrane. The dissolved gas permeates into the PDMS membrane. On the other side of the membrane, the permeated gas is desiccated by the drying box, then enters the gas chamber through the needle valve 1 and the filter, and finally returns to the PDMS membrane module through the filter, the needle valve 2 and the gas pump. Thus, water/gas separation and enrichment can be completed. The needle valve permits a flow rate of approximately 50 mL/min.

The third part, i.e., the gas detection unit, includes a control circuit (DSP, TMS320C6748, Texas Instruments, Dallas, TX, USA), a DFB laser (NLK1L5GAAA, NEL, Yokohama, Japan), a semiconductor optical amplifier (SOA, BOA1080P, Throlabs, USA), an isolator (PIISO-1600-D-L-05-FA, Qinghe Photonics, Shenzhen, China), an optical resonator and a photoelectric detector (GPD, GAP1000FC, GPD Optoelectronics, Salem, MA, USA). Sawtooth signal and step signal generated by the control circuit are combined to drive the laser to achieve laser beam resonance enhancement inside the optical resonator. When the enhanced intracavity laser power, monitored by the detector, exceeds a certain threshold, the incident laser is rapidly cut off using the TTL driven SOA to generate ring-down signal. With the ring down signal recorded, a fast single exponential fitting is performed to calculate the ring down time. After 20 recordings of ring-down time for a single longitudinal mode, the step voltage is reset to match the laser to next longitudinal mode until the whole absorption line of CO_2_ is covered. With the averaged absorption spectra, the CO_2_ absorbance and concentration are calculated by spectral fitting algorithm. Finally, the concentration of the seawater side gas is retrieved according to the exponential fitting in the unbalanced situation.

### 3.1. Absorption Line Selection

**Figure 3:**
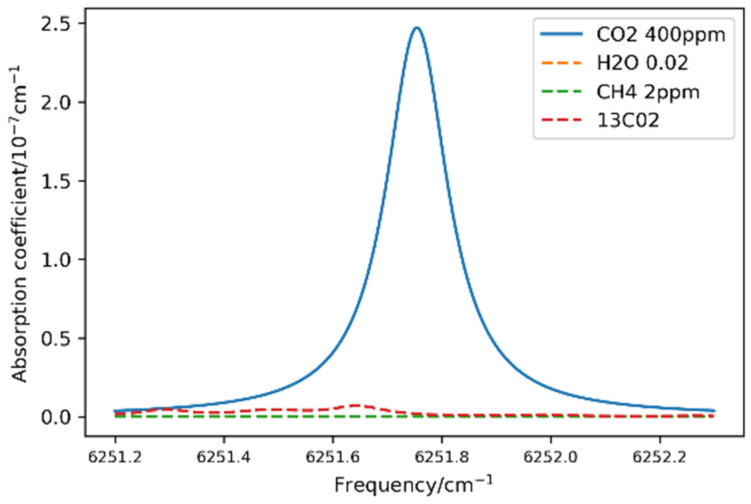
Simulation of CO_2_ absorption feature and background interference from of ^13^CO_2_, H_2_O vapor and CH_4_.

Appropriate absorption line with high absorption intensity and less background interference can benefit the dissolved CO_2_ measurement with high signal-to-noise ratio. Considering the application condition in seawater dissolved gas measurement, absorption spectra of CO_2_, ^13^CO_2_, H_2_O and CH_4_ within 1599.4–1599.7 nm is simulated based on the HITRAN database [30] (temperature: 296 K, gas pressure: 1.01 × 10^5^ Pa, CO_2_ = 400 ppm, ^13^CO_2_ =, H_2_O = 2% and CH_4_ = 2 ppm). The simulation results, shown in Figure 3, illustrate that CO_2_ absorption coefficient is 2.5 ×10^−7^ cm^−1^ with negligible interference. Thus absorption transition R(36) at 6251.761 cm^−1^ is selected for the following CO_2_ measurement.

### 3.2. Optical Resonator Design

**Figure 4:**
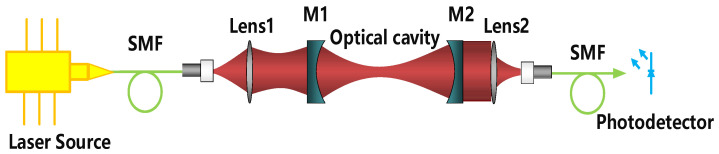
Framework of the optical resonant cavity, SMF: Single mode fiber; M1 and M2: cavity mirrors. Lens1 is a resonant cavity mode matching lens for mode matching of a laser-beam to a high finesse optical cavity. Lens2 is the light converging lens for coupling light into fiber.

The optical resonator is shown in Figure 4. Two identical cavity mirrors M1 and M2 (Layertec) have a diameter of 12.7 mm, a radius of curvature of 1000 mm and a reflectivity of higher than 99.99% (@1500~1700 nm). Lens1 and Lens2 are adjustable focus aspherical collimators (CFC-8X-C, Throlabs, Newton, NJ, USA). To suppress the high-order modes, the laser beam is spatially filtered using a single-mode fiber before illuminating on the photodetector, achieving a high-order modes suppression ratio of better than 100:1. It should be noted that, most of the optical and mechanical components of the optical cavity are fixed with structural adhesive to adapt to the extreme marine environment and improve the system stability.

### 3.3. Longitudinal Mode Matching and Spectral Scanning

**Figure 5:**
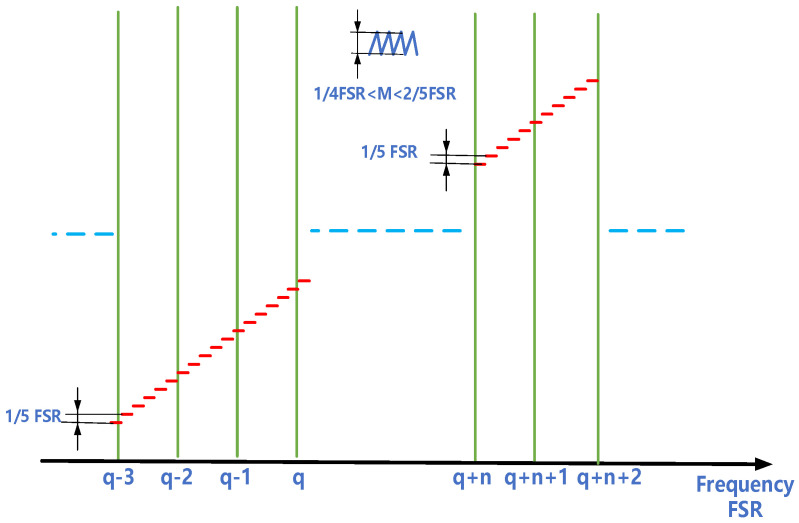
Schematic of the stepwise spectral scanning.

The physical distance between the two cavity mirrors is 420 mm, corresponding to a free spectral range (FSR) of 0.0119 cm^−1^. For each measurement using the continuous-wave CRDS system, the laser frequency is adjusted to resonate with one cavity mode. Since the cavity length remains stable during the measurement, the longitudinal cavity modes are used as the frequency reference to depict spectral absorption curve. To avoid potential spectral distortion from the longitudinal mode leakage during the spectral scanning process, we propose a strategy of longitudinal mode matching in a stepwise manner shown in Figure 5, and the step size is set as 1/5 FSR. Ideally, when the laser resonates with the longitudinal cavity mode q, 5 steps can rematch laser and the resonant cavity. However, it can be hardly realized due to the laser wavelength drift and cavity vibration. Differently, a high frequency sawtooth modulation (amplitude, 1/4FSR ≤ M ≤ 2/5FSR) is simultaneously superimposed on the step signal to ensure the resonance of each cavity mode with the laser. In the implementation of this strategy, each longitudinal mode resonates at least once in 5-step scanning, and at least one step does not resonate. The discrete spectrum can be obtained by taking the non-resonant step as a marker.

## 4. Experimental Results

### 4.1. Sensor Performance

**Figure 6:**
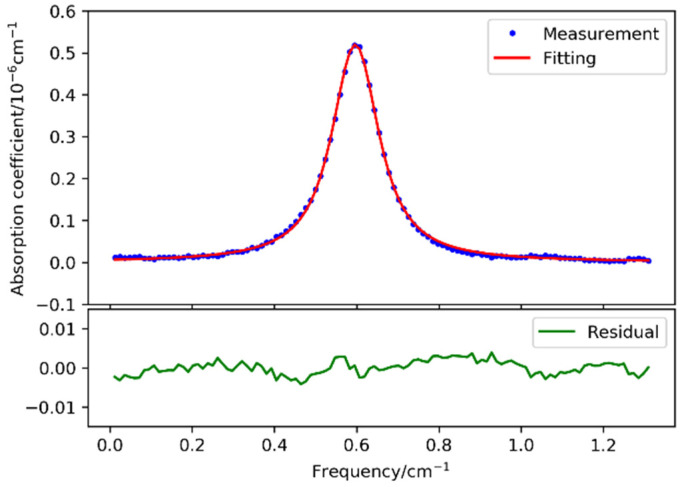
Comparison of the measured CO_2_ spectral data and Voigt fitting curve.

After assembling the sensor, calibrated CO_2_ sample with a certain concentration of 885 ppm (uncertainty, 1%) was sealed inside the gas chamber. The absorption spectrum of CO_2_ is obtained using the method described in Section 3.3. Voigt spectrum fitting was performed using a Python LMFIT-based program. Figure 6 presents a direct comparison between raw spectrum data of and the fitting curve, and a residual error of 1.5 × 10^−9^ cm^−1^. The SNR is calculated to be 500, corresponding to a detection limit of 1.8 ppm.

**Figure 7:**
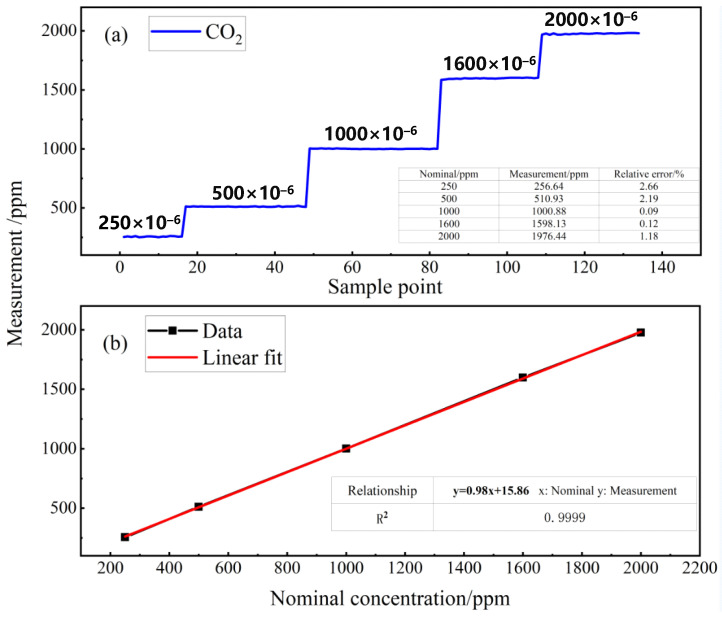
(**a**) Measured results of CO_2_ samples ranging from 250 ppm to 2000 ppm and (**b**) a linear fit to the measured data.

CO_2_ samples with different concentration of 2000 ppm, 1600 ppm, 1000 ppm, 500 ppm and 250 ppm were generated by diluting the calibrated 10000 ppm CO_2_ with pure N_2_. The uncertainty for above samples is 1%, mainly introduced by the dilution system. The mass flow meter was used to control the inlet flow rate at 50 mL/min. A continuous measurement of each CO_2_ sample was performed over 45 min and the experimental results are shown in Figure 7a.

A linear fit, shown in Figure 7b, was thus performed to the measured data and the R-square value was calculated to be 0.9999, illustrating a good linear response to the CO_2_ concentration from 250 ppm to 2000 ppm. The relative error of the measured values of the five groups of standard gases is less than 2.66%, which is consistent with the uncertainty of the standard gases. 

**Figure 8:**
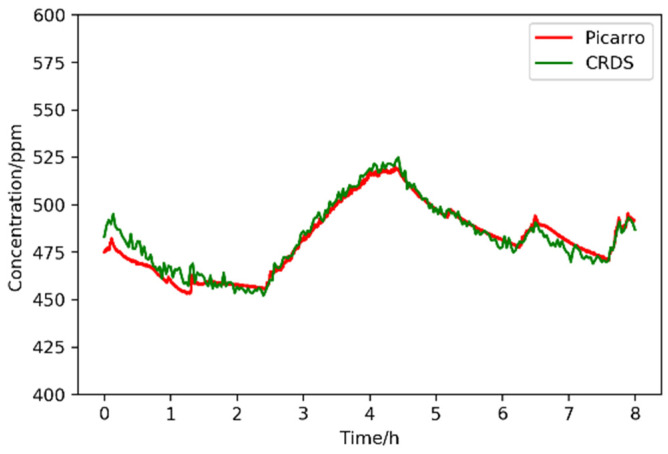
Comparison of this sensor to one commercial CRDS instrument (G2201-i, Picarro, Santa Clara, CA, USA).

To prove the feasibility, a long-term (8 h) comparison experiment was carried out between this sensor and one commercial land-based instrument CRDS instrument (G2201-i, Picarro). The gas pipelines of G2201-i and this sensor were connected together to guarantee the same the analyte was simultaneously and separately measured. The comparison result, shown in Figure 8, demonstrates that the measured CO_2_ concentration and its variation trend are consistent with each other and the maximum relative difference is within 1.3%.

### 4.2. Verification of Exponential Fit Retrieval Method for Dissolved CO_2_ Concentration

**Figure 9:**
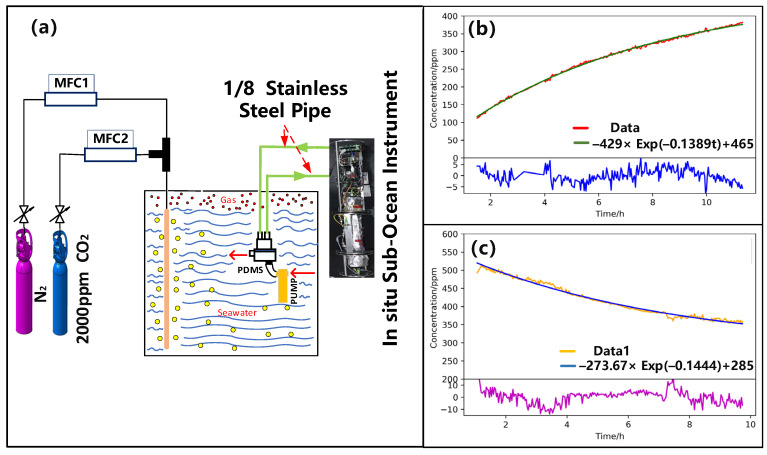
(**a**) Schematic of sample solution preparation and dissolved CO_2_ measurement system. Real-time concentration measurements when CO_2_ concentration in the cavity is (**b**) lower and (**c**) higher than the dissolved CO_2_, respectively.

Sample solutions are prepared by mixing 2000 ppm CO_2_ and pure N_2_ (purity: 99.99%) using two mass flow meters (AST10-DLCMX-100C-025-A2B2-4VE, Asert, Franklin, MA, USA), as shown in Figure 9a. A submersible pump (SBE-5T, SEA-BIRD, Bellevue, WA, USA) is used to continuously cycle the solution through the PDMS module to separate dissolved CO_2_ to be measured. PDMS module is connected to the chamber of in-situ measurement system via a stainless tube. After 12-h measurement, the exponential model combined with Levenberg Marquardt (LM) algorithm is performed on the measured data. Figure 9b depicts the results with a R-square of 99.84%, when gas-phase CO_2_ concentration in the chamber is lower than the dissolved CO_2_. Figure 9c depicts the results with a R-square of 98.71% when gas-phase CO_2_ concentration in the chamber is higher than the dissolved CO_2_. The coefficient $\frac{D_{G}S_{G}A}{lV}$, independent from the gas concentration, inside and outside the membrane are 0.1389 and 0.1444, respectively. The coefficients of two different situations are consistent with each other with a small difference of 3.8%. Thus, the method of exponential fit for dissolved gas concentration proves to be validated.

### 4.3. In-Situ Detection of Dissolved CO_2_ in Seawater

**Figure 10:**
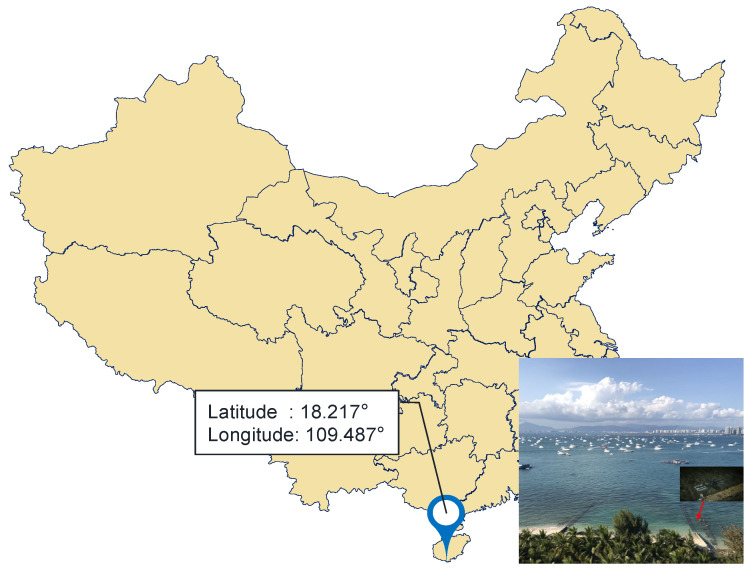
The in-situ test site is located at 18.217° north latitude and 109.487° east longitude.

From 6 to 7 March 2021, an in-situ observation was carried out near the coast of Sanya Institute of Deep Sea, Chinese Academy of Sciences, Sanya, Hainan Province. Figure 10 shows the observation location.

**Figure 11:**
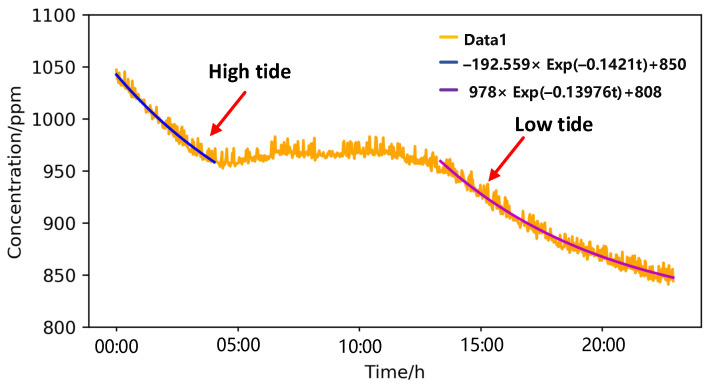
The result of In-situ measurements near coast.

At the beginning of measurement, about 1100 ppm CO_2_ was filled in the cavity to quickly balance the concentration inside and outside the membrane. Figure 11 shows the observation result, the whole measurement is divided into two unstable parts and one stable part. In the first unstable part (3 h), the measured concentration was higher than that of seawater dissolved CO_2_ due to the presence of 1100 ppm CO_2_ in the measurement chamber, and the CO_2_ diffused from the measurement chamber into seawater. The concentration of seawater dissolved CO_2_ is calculated to be 850 ppm. In the second stable part (9 h), the gas concentration inside and outside the membrane remained equal, and the dissolved gas concentration fluctuated between 950 ppm to 980 ppm. In the third unstable part (8 h), the concentration of dissolved CO_2_ decreased and the CO_2_ in the cavity continued to diffuse into seawater. The concentration of dissolved CO_2_ in the water is calculated to be 808 ppm. Interestingly, these measured dissolved CO_2_ concentrations are much higher than the atmospheric CO_2_ concentration, about 400 ppm, mainly because of a number of yachts cruising near the coast and the tide phenomenon.

## 5. Conclusions

We develop a fiber-integrated in-situ dissolved CO_2_ sensor using CRDS, in which a PDMS membrane is employed for water/gas separation and enrichment, and an exponential regression model is proposed for fast dissolve CO_2_ retrieval. The model feasibility is verified by performing a comparison test under two different situations, i.e., measurement chamber CO_2_ concentration is higher and lower than the dissolved CO_2_ concentration. Both their R-square of fitting are better than 98.5% and the difference of the two individually measured PDMS membrane coefficient $\frac{D_{G}S_{G}A}{lV}$ is only 3.8%. The whole absorption spectrum of CO_2_ can be obtained within 90 s and a detection sensitivity of 1.8 ppm has been achieved. A near coast in-situ measurement has been implemented over 24 h and provided regular fluctuation of dissolved CO_2_ concentrations, which is due to the tide phenomenon. Future efforts will be made to improve the corrosion resistance ability by using titanium alloy stainless steel as the pressured chamber material and to improve the gas separation efficiency by increasing the membrane surface area. Therefore, the developed senor could act as a promising tool to achieve high-precision detection of dissolved gas in seawater and then support the investigation on the ocean, such as vertical dissolved CO_2_ profile as deep as 4500 m and long-term dissolved CO_2_ monitoring under deep-sea extreme environment window, e.g., hydrothermal and cold spring.

## Data Availability

The data that support the plots within this paper are available from the corresponding author on request basis.
